# Application of automated peritoneal dialysis in urgent-start peritoneal dialysis patients during the break-in period

**DOI:** 10.1007/s11255-018-1785-1

**Published:** 2018-01-16

**Authors:** Shengmao Liu, Xiaohua Zhuang, Min Zhang, Yanfeng Wu, Min Liu, Sibo Guan, Shujun Liu, Lining Miao, Wenpeng Cui

**Affiliations:** Department of Nephrology, Second Hospital, Jilin University, 218 Ziqiang Street, Changchun, 130041 Jilin China

**Keywords:** Peritoneal dialysis, Break-in period, Automated peritoneal dialysis, Continuous ambulatory peritoneal dialysis

## Abstract

**Objective:**

Whether automated peritoneal dialysis (APD) is a feasible strategy for urgent-start peritoneal dialysis (PD) therapy during the break-in period remains unclear. This study was conducted to compare the efficacy as well as complications among three PD modes during the break-in period.

**Methods:**

Ninety-six patients treated with urgent-start PD after catheterization were retrospectively analyzed. Patients were divided into three groups, incremental continuous ambulatory PD (CAPD) group (*n* = 26); APD group (*n* = 42); and APD–CAPD group (*n* = 28). Clinical parameters at the end of the break-in period and 1 month after the initiation of PD treatment were collected and analyzed.

**Results:**

Compared with the traditional incremental CAPD, APD and APD–CAPD were superior as they could effectively remove small-molecule uremic toxins and correct electrolyte imbalance (*P* < 0.05), while did not increase the incidence of early complications during the break-in period (*P* > 0.05). However, APD led to a significant decline in albumin and pre-albumin, as compared with APD–CAPD and CAPD (*P* < 0.05). A PD strategy consisting 6 days of APD and 3 days of CAPD showed a great advantage in preventing excessive protein loss. There were no significant differences in all tested biochemical parameters among the three groups at 1 month after treatment (all *P* > 0.05).

**Conclusion:**

Application of APD for urgent-start PD during the break-in period is feasible. A combination of APD and CAPD regimens seems to be a more reasonable mode.

## Introduction

Peritoneal dialysis (PD) is one of the common renal replacement therapies for patients with end-stage renal disease (ESRD). The break-in period refers to the time between catheter insertion and routine catheter use. The treatment strategy used during the break-in period allows patients to adapt to the dialysis process. Patients usually undergo the PD break-in period of 2 weeks after catheterization [1]. However, there are some patients needing an urgent-start PD immediately after PD catheter insertion. In order to reduce the incidences of mechanical complications caused by urgent-start of PD treatment such as peritoneal fluid leakage and hernia, an incremental initiation of continuous ambulatory peritoneal dialysis (CAPD) is traditionally applied over the break-in period by gradual introduction of dialysate exchanges from a small-dose to full-dose therapy (e.g., from 500–800 to 2000 mL each session) [2]. Considering insufficient volume of dialysate exchanges, some researchers advocate intermittent PD (IPD) by increased times of dialysis fluid exchange [3]. However, frequent dialysis exchange will not only increase the workload of healthcare workers, but also incur increased risk of infection.

With the advent of automatic peritoneal dialysis (APD) machine, PD can be carried out automatically by filling and draining the dialysate, and fewer connections and disconnections could potentially reduce the risk of peritonitis [4]. In recent years, APD has been reported to be used for urgent-start PD treatment [5–7]. However, no consensus has been reached on the optimal PD mode during the break-in period, especially for the dose of PD.

In this study, a total of 96 ESRD patients who treated with urgent-start PD after catheterization were retrospectively analyzed. The aim of the study was to evaluate the different PD modes during the break-in period and to establish an appropriate treatment strategy for patients with urgent-start PD.

## Materials and methods

### Participants

The ESRD patients who treated with urgent-start PD after catheterization in the Second Hospital of Jilin University from October 2013 to July 2017 were enrolled. Inclusion criteria included (1) age between 18 and 85 years old, male or female; (2) diagnosis of ESRD; (3) urgent-start PD on 1–3 days after catheterization; (4) nine-day treatment during break-in period. The following criteria were used to exclude patients from this study: acute renal failure; hemodialysis during the break-in period; hemorrhagic complications after catheterization; systemic co-morbidities such as malignancies, systemic infection, cirrhosis and congestive heart failure.

### PD prescription

All patients received the placement of polyester double-cuff straight Tenckhoff catheter by experienced surgeons in accordance with standard operating procedures. The implantation of PD catheter was performed by an open surgery. Briefly, a left paramedian incision was made at 9–13 cm above the pubic symphysis. Subcutaneous tissue was carefully detached to reach anterior sheath of the rectus muscle, and the anterior rectus sheath was incised (2–4 cm in length). And then the posterior rectus abdominis sheath was opened and cut to expose the peritoneum after blunt detachment. The peritoneum was then incised to create a small opening. With the help of a guide wire, the PD catheter was placed into the peritoneal cavity of the abdomen. All operations were performed by the same team of clinicians.

Patients were divided into three groups according to PD prescription during the break-in period, namely APD group (*n* = 42, APD for 9 days), APD–CAPD group (*n* = 28, APD for 6 days followed by CAPD for 3 days) and CAPD group (*n* = 26, incremental CAPD for 9 days). The regimen in the APD group included 9 fill/drain cycles of 650 mL over 48 min and one long overnight dwell for the first 3 days, 8 cycles of 1000 mL over 48 min and a dwell overnight during 4–6 days, and 6 cycles of 1500 mL over 48 min and a dwell overnight during 7–9 days. The regimens in the CAPD were 4 cycles of 500–800 mL over 3–4 h and dwell overnight for the first 3 days, 4 cycles of 1000 mL over 3–4 h and dwell overnight during 4–6 days, and 4 cycles of 1500 mL over 4 h and dwell overnight during 7–9 days. Patients in the APD–CAPD group were treated with APD regimen that was consistent with the APD group for the first 6 days, and CAPD during 7–9 days (4 cycles of 1500 mL over 4 h and dwell overnight).

### Data collection

The laboratory data, blood pressure, estimated glomerular filtration rate (eGFR), PD complications (PD-associated peritonitis, catheter-related infection, mechanical complications, etc.) were collected before and after the break-in period as well as at a month after the initiation of PD treatment. eGFR was calculated by CKD-EPI formula among patients who initiated PD; while for the patients who underwent PD, residual kidney GFR was estimated by the formula: (renal urea clearance rate + renal creatinine clearance rate)/2. A routine peritoneal equilibration test was performed at 1 month after catheterization.

### Statistical analysis

The SPSS 19.0 statistical software package was used for statistical analysis. Continuous data were expressed as mean ± standard deviation (SD), and categorical data were expressed as absolute value and percentage. Continuous data were analyzed by the *t* test and analysis of variance with least significant difference test to evaluate differences among groups. Numeration data were analyzed with Chi-square test. *P* < 0.05 was considered statistically significant.

## Results

### Demographic and baseline characteristics of subjects

A total of 96 ESRD patients who treated with urgent-start PD after catheterization were enrolled, including 50 (52.1%) males and 46 (47.9%) females, with an average age of 53.91 ± 1.54 (range 22–77) years old. There were no significant differences in gender distribution, age, blood pressure, proportion of diabetic nephropathy, biochemical indicators, and the use of medication for hypertension control, improving anemia and decreasing phosphate levels during the break-in period among the three groups (all *P* > 0.05, Table 1).

**Table 1:** Demographic and baseline characteristics of the subjects

	APD group (*n* = 42)	APD–CAPD group (*n* = 28)	CAPD group (*n* = 26)	*P* value^†^
Male, *n* (%)	23 (54.8)	14 (50.0)	13 (50.0)	0.898
Age (years)	51.88 ± 13.49	55.00 ± 16.61	55.74 ± 9.77	0.527
Systolic blood pressure (mmHg)	152.70 ± 16.90	147.70 ± 18.00	154.90 ± 20.89	0.418
Diabetic nephropathy, *n* (%)	15 (35.7)	12 (42.8)	8 (30.8)	0.648
Medication, *n* (%)				
Antihypertensive agents	30 (71.4)	22 (67.9)	19 (73.1)	0.795
Anemia-improving agents*	31 (73.8)	22 (78.6)	18 (69.2)	0.737
Phosphorus-reducing agents	22 (52.4)	14 (50.0)	15 (57.7)	0.845
Blood urea nitrogen (mmol/L)	25.05 ± 8.97	22.66 ± 9.12	21.29 ± 7.17	0.195
Creatinine (umol/L)	853.40 ± 233.15	811.10 ± 225.99	824.30 ± 279.70	0.761
Uric acid (umol/L)	445.80 ± 108.00	437.70 ± 126.78	434.60 ± 88.64	0.907
iPTH (pg/mL)	418.90 ± 193.32	447.90 ± 165.40	445.90 ± 228.64	0.845
Serum potassium (mmol/L)	4.72 ± 0.77	4.60 ± 0.72	4.63 ± 0.75	0.803
Serum calcium (mmol/L)	2.02 ± 0.27	2.08 ± 0.23	2.11 ± 0.25	0.323
Serum phosphorus (mmol/L)	1.99 ± 0.73	1.89 ± 0.59	1.92 ± 0.71	0.805
Serum sodium (mmol/L)	140.20 ± 2.95	140.10 ± 3.53	140.90 ± 5.06	0.735
CO2-CP (mmol/L)	22.24 ± 2.39	21.73 ± 3.07	22.58 ± 3.04	0.532
Albumin (g/L)	35.13 ± 6.12	33.87 ± 5.44	32.93 ± 5.52	0.298
Pre-albumin (mg/L)	301.20 ± 76.53	291.70 ± 52.59	289.20 ± 75.21	0.751
Total protein (g/L)	65.06 ± 11.19	65.56 ± 9.18	64.15 ± 8.71	0.872
Fasting plasma glucose (mmol/L	5.61 ± 1.55	5.65 ± 1.78	5.57 ± 1.06	0.984
Hemoglobin (g/L)	80.69 ± 13.43	82.54 ± 12.46	81.35 ± 14.67	0.855
eGFR (mL/min/1.73 m^2^)^#^	5.71 ± 2.10	5.52 ± 1.69	6.40 ± 2.29	0.312
Urine volume (mL)	1352.00 ± 303.57	1381.00 ± 446.44	1581.00 ± 545.54	0.149

APD, automatic peritoneal dialysis; CAPD, continuous ambulatory peritoneal dialysis; CO2-CP, carbon dioxide combining power; eGFR, estimated glomerular filtration rate; iPTH, intact parathyroid hormone

*Included erythropoiesis-stimulating agents, iron, folic acid and vitamin B12

^#^CKD-EPI formula was used to calculate eGFR

^†^The *P* values were compared among the three groups by one-way ANOVA

### Comparison of changes in parameters among groups after treatment during the break-in period

APD and APD–CAPD were able to clear small-molecule toxins (creatinine, blood urea nitrogen and uric acid) and electrolytes (potassium and phosphorus), by comparison of biochemical indicators before and after treatments (*P* < 0.05, Table 2). For CAPD, only clearance of blood urea nitrogen and potassium was achieved to a statistical significance level (*P* < 0.05).

**Table 2:** Parameters after treatment over the break-in period

	APD group (*n* = 42)	*P*	APD–CAPD group (*n* = 28)	*P*	CAPD group (*n* = 26)	*P*
Toxins						
Blood urea nitrogen (mmol/L)	14.41 ± 5.68	0.000	13.51 ± 4.03	0.000	16.40 ± 5.43	0.008
Creatinine (umol/L)	657.80 ± 193.71	0.000	627.30 ± 154.41	0.001	711.00 ± 258.94	0.136
Uric acid (umol/L)	370.60 ± 73.48	0.000	357.80 ± 107.32	0.014	404.50 ± 87.20	0.223
Electrolytes						
Serum potassium (mmol/L)	3.80 ± 0.50	0.000	3.75 ± 0.51	0.000	4.17 ± 0.51	0.012
Serum calcium (mmol/L)	2.12 ± 0.25	0.069	2.17 ± 0.25	0.154	2.23 ± 0.31	0.132
Serum phosphorus (mmol/L)	1.43 ± 0.38	0.000	1.27 ± 0.27	0.000	1.68 ± 0.47	0.160
Serum sodium (mmol/L)	139.00 ± 3.75	0.094	141.20 ± 3.50	0.277	141.50 ± 3.34	0.589
CO2-CP (mmol/L)	24.51 ± 2.70	0.000	24.93 ± 3.75	0.001	24.48 ± 2.01	0.010
Nutritional indicators						
Albumin (g/L)	29.81 ± 4.85	0.000	31.33 ± 5.32	0.083	32.32 ± 5.38	0.691
Pre-albumin (mg/L)	268.60 ± 87.23	0.072	299.60 ± 107.41	0.728	302.20 ± 83.05	0.556
Total protein (g/L)	58.30 ± 8.70	0.003	61.72 ± 8.83	0.117	60.19 ± 7.18	0.080
Others						
FPG (mmol/L)	5.32 ± 0.87	0.302	5.46 ± 0.92	0.622	5.58 ± 1.27	0.983
Hemoglobin (g/L)	81.26 ± 15.34	0.856	83.00 ± 11.44	0.885	85.19 ± 13.86	0.336

APD, automatic peritoneal dialysis; CAPD, continuous ambulatory peritoneal dialysis; CO2-CP, carbon dioxide combining power; FPG, fasting plasma glucose. *t* test for comparing the value before PD and after break-in period, *P* < 0.05

We next compared the efficacy of three PD modes in the break-in period (Table 3). APD and APD–CAPD were superior to CAPD in clearance of serum creatinine, blood urea nitrogen and uric acid (*P* < 0.05). In addition, APD and APD–CAPD were more potent in reduction of potassium and phosphorus than CAPD only (*P* < 0.05). However, there was no significant difference in calcium, sodium and carbon dioxide combining power changes after treatment among groups (*P* > 0.05). APD led to a significant decline in albumin and pre-albumin, as compared with APD–CAPD and CAPD (*P* < 0.05). There was no significant difference in albumin and pre-albumin reduction between the APD–CAPD group and CAPD group (*P* > 0.05).

**Table 3:** Comparison of changes in parameters after treatment over the break-in period among the three groups

	APD group (*n* = 42)	APD–CAPD group (*n* = 28)	CAPD group (*n* = 26)	*P* ^a^	*P* ^b^	*P* ^c^
Toxins						
Blood urea nitrogen (mmol/L)	− 10.64 ± 6.66	− 9.15 ± 6.78	− 4.89 ± 3.57	0.367	0.000	0.006
Creatinine (umol/L)	− 195.50 ± 114.84	− 183.80 ± 161.41	− 113.50 ± 61.73	0.723	0.042	0.001
Uric acid (umol/L)	− 75.14 ± 67.62	− 79.86 ± 56.42	− 30.12 ± 56.83	0.762	0.006	0.002
Electrolytes						
Serum potassium (mmol/L)	− 0.91 ± 0.78	− 0.86 ± 0.72	− 0.46 ± 0.53	0.763	0.012	0.027
Serum calcium (mmol/L)	0.11 ± 0.32	0.09 ± 0.31	0.12 ± 0.23	0.879	0.860	0.743
Serum phosphorus (mmol/L)	− 0.57 ± 0.61	− 0.61 ± 0.63	− 0.24 ± 0.38	0.752	0.017	0.011
Serum sodium (mmol/L)	− 1.25 ± 4.83	1.03 ± 4.63	0.65 ± 3.01	0.053	0.078	0.720
CO2-CP (mmol/L)	2.27 ± 2.95	3.20 ± 3.87	1.91 ± 3.92	0.261	0.665	0.968
Nutritional indicators						
Albumin (g/L)	− 5.30 ± 4.70	− 2.54 ± 4.37	− 0.60 ± 5.12	0.016	0.000	0.140
Pre-albumin (mg/L)	− 32.64 ± 66.39	7.89 ± 90.43	13.04 ± 80.97	0.035	0.014	0.827
Total protein (g/L)	− 6.76 ± 5.78	− 3.87 ± 6.66	− 3.96 ± 4.98	0.059	0.045	0. 958
Others						
FPG (mmol/L)	− 0.28 ± 1.62	− 0.19 ± 1.71	0.01 ± 1.56	0.813	0.469	0.665
Hemoglobin (g/L)	0.57 ± 10.50	0.46 ± 13.03	3.85 ± 7.55	0.970	0.172	0.253

APD, automatic peritoneal dialysis; CAPD, continuous ambulatory peritoneal dialysis; CO2-CP, carbon dioxide combining power

^a^Comparison between the APD group and APD–CAPD group; ^b^comparison between the APD group and CAPD group; ^c^comparison between the APD–CAPD group and CAPD group

### Comparison of PD-related complications of three PD modes during the break-in period

There were no differences in the incidences of PD-related complications among three groups, including catheter migration, PD fluid leakage, abdominal pain, abdominal distention, catheter obstruction, PD- or catheter-related peritonitis during the break-in period (all *P* > 0.05, Table 4).

**Table 4:** Comparison of PD-related complications over the break-in period

Complications, *n* (%)	APD group (*n* = 42)	APD–CAPD group (*n* = 28)	CAPD group (*n* = 26)	Total (*n* = 98)	*P* value
Catheter migration	4 (9.5)	3 (10.7)	2 (7.7)	9 (9.2)	0.929
PD fluid leakage	2 (4.8)	1 (3.6)	1 (3.8)	4 (4.1)	0.966
Abdominal pain	3 (7.1)	3 (10.7)	2 (7.7)	8 (8.2)	0.861
Abdominal distention	2 (4.8)	1 (3.6)	1 (3.8)	4 (4.1)	0.966
Catheter obstruction	1 (2.4)	1 (3.6)	1 (3.8)	3 (3.1)	0.932
PD-related peritonitis	1 (2.4)	0	1 (3.8)	2 (2.0)	0.603
Catheter-related peritonitis	1 (2.4)	1 (3.6)	2 (7.7)	4 (4.1)	0.557

PD, peritoneal dialysis

### Comparison of changes in parameters among groups at a month after treatment

All the three types of PD models were able to clear toxins (creatinine, blood urea nitrogen, uric acid and iPTH) and improve electrolyte disorders (potassium, calcium, phosphorus and CO2-CP), by comparison of biochemical indicators before and after treatment (*P* < 0.05, Table 5).

**Table 5:** Parameters at a month after treatment

	APD group (*n* = 42)	*P*	APD–CAPD group (*n* = 28)	*P*	CAPD group (*n* = 26)	*P*
Toxins						
Blood urea nitrogen (mmol/L)	14.07 ± 4.76	0.000	13.42 ± 4.79	0.001	11.54 ± 3.71	0.000
Creatinine (umol/L)	694.40 ± 186.75	0.003	610.20 ± 185.66	0.004	638.10 ± 211.03	0.010
Uric acid (umol/L)	372.00 ± 79.12	0.002	358.30 ± 113.98	0.045	382.50 ± 82.38	0.035
iPTH (pg/mL)	321.20 ± 180.91	0.044	306.3 ± 138.85	0.014	305.3 ± 159.74	0.017
Electrolytes						
Serum potassium (mmol/L)	4.22 ± 0.63	0.005	4.15 ± 0.46	0.029	4.39 ± 0.59	0.210
Serum calcium (mmol/L)	2.25 ± 0.27	0.000	2.41 ± 0.23	0.000	2.30 ± 0.17	0.002
Serum phosphorus (mmol/L)	1.58 ± 0.39	0.006	1.42 ± 0.40	0.008	1.46 ± 0.43	0.007
Serum sodium (mmol/L)	142.10 ± 3.15	0.011	143.70 ± 2.77	0.001	142.30 ± 2.83	0.209
CO2-CP (mmol/L)	25.37 ± 2.68	0.000	24.66 ± 2.50	0.002	25.52 ± 3.10	0.001
Nutritional indicators						
Albumin (g/L)	38.12 ± 6.05	0.042	38.75 ± 3.97	0.003	37.88 ± 3.33	0.000
Pre-albumin (mg/L)	375.40 ± 95.85	0.001	361.80 ± 59.67	0.000	339.40 ± 105.90	0.056
Total protein (g/L)	67.96 ± 7.41	0.213	70.64 ± 7.27	0.065	68.50 ± 5.86	0.043
Others						
FPG (mmol/L)	6.25 ± 1.87	0.111	7.34 ± 3.38	0.035	6.91 ± 2.25	0.009
Hemoglobin (g/L)	107.30 ± 15.62	0.000	112.10 ± 11.94	0.000	110.20 ± 18.98	0.000
GFR (mL/min/1.73 m^2^)^#^	4.08 ± 2.03	0.003	4.08 ± 1.70	0.005	5.26 ± 2.37	0.114
Urine volume (mL)	1190.00 ± 531.33	0.153	1308.00 ± 494.24	0.594	1376.00 ± 420.60	0.181

APD, automatic peritoneal dialysis; CAPD, continuous ambulatory peritoneal dialysis; CO2-CP, carbon dioxide combining power; FPG, fasting plasma glucose; GFR, glomerular filtration rate. *t* test for comparing the value before PD and after break-in period, *P* < 0.05

^#^Residual kidney GFR was estimated by the formula: (renal urea clearance rate + renal creatinine clearance rate)/2

There were no significant differences in all tested biochemical parameters among groups at 1 month after treatment (all *P* > 0.05, Table 6).

**Table 6:** Comparison of changes in parameters among groups at a month after treatment

	APD group (*n* = 42)	APD–CAPD group (*n* = 28)	CAPD group (*n* = 26)	*P* value
Toxins				
Blood urea nitrogen (mmol/L)	− 10.13 ± 7.98	− 8.15 ± 10.45	− 8.65 ± 7.09	0.689
Creatinine (umol/L)	− 172.50 ± 100.23	− 195.60 ± 142.75	− 159.90 ± 177.34	0.731
Uric acid (umol/L)	− 61.77 ± 81.87	− 47.13 ± 99.23	− 50.84 ± 74.94	0.817
iPTH (pg/ml)	− 97.73 ± 159.48	− 141.40 ± 82.40	− 140.70 ± 124.94	0.409
Electrolytes				
Serum potassium (mmol/L)	− 0.53 ± 0.93	− 0.34 ± 0.87	0.01 ± 0.65	0.059
Serum calcium (mmol/L)	0.26 ± 0.24	0.30 ± 0.26	0.19 ± 0.31	0.434
Serum phosphorus (mmol/L)	− 0.59 ± 0.51	− 0.73 ± 0.63	− 0.60 ± 0.70	0.728
Serum sodium (mmol/L)	1.71 ± 3.41	3.30 ± 5.14	1.32 ± 4.78	0.343
CO2-CP (mmol/L)	2.57 ± 3.64	2.49 ± 2.66	3.12 ± 4.50	0.823
Nutritional indicators				
Albumin (g/L)	3.37 ± 4.54	4.21 ± 4.11	4.89 ± 4.50	0.443
Pre-albumin (mg/L)	62.33 ± 97.06	45.44 ± 81.62	43.00 ± 74.10	0.669
Total protein (g/L)	3.27 ± 7.41	6.02 ± 4.32	6.38 ± 6.59	0.169
Others				
Fasting plasma glucose (mmol/L)	0.45 ± 2.67	1.72 ± 2.30	1.42 ± 1.77	0.142
Hemoglobin (g/L)	26.26 ± 14.33	32.47 ± 14.08	28.43 ± 21.58	0.475
GFR (mL/min/1.73 m^2^)	− 1.63 ± 1.63	− 1.44 ± 1.60	− 1.14 ± 2.65	0.679
Urine volume (mL)	− 161.70 ± 528.45	− 72.92 ± 469.64	− 195.50 ± 550.74	0.706

CO2-CP, carbon dioxide combining power; iPTH, intact parathyroid hormone; GFR, glomerular filtration rate

^#^Residual kidney GFR was estimated by the formula: (renal urea clearance rate + renal creatinine clearance rate)/2

### Peritoneal equilibration tests for three PD modes

There were no significant differences in the ratios of number of patients with high peritoneal transport to those with low transport among groups (*P* > 0.05, Table 7).

**Table 7:** Results of peritoneal equilibration tests for three PD modes

	APD group	APD–CAPD group	CAPD group	*P*
High transport/low transport (*n*/*n*)^a^	24/18	15/13	15/11	0.943

^a^High transport: high transport + high average transport; low transport: low transport + low average transport

## Discussion

Peritoneal dialysis is an effective method of removing of fluid and various sizes of solute molecules. Our results showed that APD and APD–CAPD were able to equivalently clear small-molecule toxins, which was superior to CAPD alone. The results were consistent with other studies [8–10]. The reasons were probably because the former was related to larger volume of dialysate exchanges, shorter retention time and more cycles. However, some researchers denied the possibility of improved clearance of small-molecule toxins by increased retention duration, which on the contrary would lead to the retention of toxic components in dialysate and decreased solute clearance rate [11]. In this study, high dose of APD yielded a superior small-molecule clearance rate, but similar intermediate- and large-molecule clearance rates in comparison with low dose of CAPD during the break-in period. These findings were similar to the previous results [10]. However, confirmation of these findings will require further investigation with a larger sample size.

Evidence has shown that the mortality rate in PD patients can be reduced by 11–47% with every increase in GFR of 5–10 L/min/1.73 m^2^ [12]. Preservation of residual renal function (RRF) at the greatest extent should be considered when selecting a HD mode. By observing 505 CAPD and 78 APD patients, Michels et al. [13] found that the risk of complete loss of renal function within the first years in APD group was twofold higher than that of CAPD group. The unfavorable outcome in RRF caused by APD may be explained by a large amount of ultrafiltration in a short period of time that can lead to renal ischemia. However, in a multicenter controlled study, no significant difference in RRF decline was found between APD and CAPD treatment during 90 days after the start of dialysis treatment [14]. Similarly, our results did not reveal a significant difference in GFR values among groups at 1 month after PD treatment. Efforts should be made to clarify the impact of different PD modes on the long-term RRF.

PD patients are at high risk of hypokalemia, with an incidence rate of 15–60% [15]. For a patient who undergoes a standard CAPD (8 L/day), approximate 40 mEq of potassium ion is removed daily, and the clearance rate is about 7–26 mL/min [16]. A study revealed increased probability of hypokalemia occurrence in patients who underwent APD at a weekly dialysis dose of 90 L or more [17]. In the present study, APD and APD–CAPD treatment led to more remarkable decline in potassium than CAPD did during the break-in period, probably due to short retention time and larger volume of dialysate exchanges in APD group.

A number of studies showed that the occurrence of hypokalemia and malnutrition are closely related [17–19]. Malnutrition is one of the most common complications of PD patients. According to the latest study, 67.84% of patients with PD have mild to moderate malnutrition and 7.07% have severe malnutrition [20]. A number of studies show that malnutrition is an important indicator of predicting the mortality of PD patients [21, 22]. Although APD achieves satisfactory clearance of small-molecule toxins, it leads to higher protein losses than CAPD due to multiple nighttime exchanges [8]. Our results supported the evidence that APD resulted in a more severe reduction of albumin and pre-albumin than CAPD during the break-in period. Interestingly, the levels of albumin and pre-albumin were not different among the three groups at a month after treatment. This probably is because APD enabled a potent clearance of toxins, which improved the appetite and digestive capacity of the patients, thereby promoting protein intake and absorption. Thus, APD–CAPD seemed to be an optimal PD mode during the break-in period, as it exhibited a high capacity of uremic toxin clearance and did not increase the risk of malnutrition as well.

Peritonitis is a serious complication of PD patients, leading to technical failure rate of up to 78% [23], rehospitalization rate of 13.5% [24] and PD peritonitis-related mortality rate of 15.2% [25]. The APD mode reduces the number of daily connections and disconnections and the chance of manual operation, thus decreasing the incidence of peritonitis [26]. A meta-analysis demonstrated that the incidence of peritonitis in APD group was decreased by 46% as compared with CAPD group [27]. In this study, the incidences of PD- and catheter-related peritonitis were 4.8, 3.6 and 11.5% in the APD group, APD–CAPD group and CAPD group, respectively, suggesting the advantage of APD over CAPD as reduced incidence of peritonitis.

Patients usually undergo a PD break-in period of 2 weeks after catheterization to improve the long-term life expectancy of the catheter and minimize the mechanical complications [28]. The incidence of catheter displacement is reported as high as 12.7–35% [29]. Immediate start of PD leads to an increased incidence of peritoneal fluid leakage (7.7%) [7]. In this study, the overall incidence rates of catheter displacement and PD fluid leakage were 9.4% (9/96) and 4.2% (4/96), revealing that urgent-start PD did not increase the occurrence of catheter displacement as well as peritoneal fluid leakage, which was consistent with previous study [5]. The low rates of mechanical complications in this study probably attributed to the incremental initiation of PD treatment which enabled a gradual increase in intra-abdominal pressure of patients.

This study was a single-center, retrospective cohort study with a relatively small sample size. Further studies with larger sample size are needed to confirm these findings.

In conclusion, compared with the traditional incremental CAPD, APD mode could effectively remove uremic toxins, correct electrolyte imbalance, while did not increase the incidence of early complications during the break-in period. A PD strategy consisting 6 days of APD and 3 days of CAPD showed a great advantage in preventing excessive protein loss. Thus, a combination of APD and CAPD regimens is recommend for patients with urgent-start PD during the break-in period.

## Abbreviations

APD: Automatic peritoneal dialysis
CAPD: Continuous ambulatory peritoneal dialysis
eGFR: Estimated glomerular filtration rate
ESRD: End-stage renal disease
IPD: Intermittent peritoneal dialysis
PD: Peritoneal dialysis
RRF: Residual renal function
SEM: Standard error of mean
